# The use of traditional and complementary medicine by cancer patients in SSA: A scoping review

**DOI:** 10.4102/hsag.v29i0.2743

**Published:** 2024-12-03

**Authors:** Ammaarah Sheik Adam, Varsha Bangalee, Frasia Oosthuizen

**Affiliations:** 1 Discipline of Pharmaceutical Sciences, College of Health Sciences, University of KwaZulu-Natal, Durban, South Africa

**Keywords:** complementary medicine, alternative medicine, traditional medicine, cancer patients, sub-Saharan Africa

## Abstract

**Background:**

The rate of traditional and complementary medicine (T&CM) use is increasing worldwide, including among cancer patients who are often willing to try alternate therapies. Despite T&CM popularity in sub-Saharan Africa (SSA), there are limited data on its use with conventional treatments.

**Aim:**

This scoping review aims to describe the prevalence of use, reasons for use, most common types of T&CM used, patient satisfaction with T&CM and disclosure of T&CM use to physicians among cancer patients in SSA.

**Methods:**

A systematic literature search was conducted for articles published from 2013 to 2022 across four databases: PubMed, ScienceDirect, Google Scholar and EBSCOhost. A scoping review approach was used to map relevant literature. Forty-six articles were assessed based on their titles and abstracts. After full-text screening, 10 articles were included.

**Results:**

Average T&CM use was calculated to be 66.7%. Reasons for T&CM use included: to improve psychological well-being, boost the body’s immunity and provide symptomatic relief. An average of 21.2% of T&CM users informed their physician. Nine articles addressed patient satisfaction with T&CM. An average of 39.1% of patients reported general T&CM satisfaction.

**Conclusion:**

The use of T&CM is common among cancer patients. It is important for healthcare providers to expand their knowledge in order to counsel patients and avoid potential hazards when combining T&CM with conventional treatments.

**Contribution:**

These findings highlight commonly used T&CM and provide insight on the portion of cancer patients informing their doctors about T&CM use. This information will help understand the attitude cancer patients have towards T&CM.

## Introduction

As defined by the World Health Organization (WHO 2019), complementary medicine (CM) is a collection of healthcare practices that fall outside the country’s conventional medicine and are not entirely incorporated into the mainstream healthcare system. The WHO states that traditional medicine (TM) refers to the knowledge, skills and practices from indigenous theories, beliefs and experiences of different cultures being used to maintain health, as well as prevent, diagnose, improve or treat physical and mental illness (WHO 2019). The WHO further merges both terms to create traditional and complementary medicine (T&CM), which includes products, practices and practitioners (WHO 2019).

Traditional and complementary medicines are being used extensively for many illnesses such as diabetes mellitus, hypertension, human immunodeficiency virus (HIV) and acquired immunodeficiency syndrome (AIDS), cancer, asthma and pain (James et al. 2018). In the African setting, specifically, it is largely made up of local herbal products or medicines and indigenous traditional healthcare practices. Several CM products and practices have also been imported, such as acupuncture and homeopathy. Sub-Saharan Africa (SSA) is one of the regions where T&CM use is popular and has become a means on which a large population rely on it to maintain health, prevent and combat diseases, as well as treat mental, physical and social disorders (James et al. 2018).

A considerable portion of South Africa’s population prefers using traditional medicine or herbal products over conventional or Western medicine as they find traditional medicine and practices more accessible, affordable and efficient while aligning with their beliefs (Monakisi 2007). According to a review done in South Africa in 2007, it was found that trade in traditional medicines in the country was worth approximately R2.9 billion per year. In South Africa alone, it is evident that the use of T&CM is vast with at least 27 million consumers (Mander, Ntuli & Diederichs 2007).

In 2010, the Allied Health Professions Council of South Africa (AHPCSA) estimated that 1% – 19% of South Africa’s population used T&CM practices, including acupuncture, aromatherapy, chiropractic, herbal medicines, homeopathy, massage therapy, naturopathy, osteopathy, therapeutic reflexology, traditional Chinese medicine and traditional Indian medicine such as Ayurveda and Unani (WHO 2019). Complementary medicine is frequently used alongside conventional treatment. Often cancer patients become depressed and have to deal with many physical and emotional aspects of the disease, leaving them open to try T&CM to aid them in their fight against cancer (Dehghan et al. 2022). This scoping review includes the following T&CM categories used in sub-Saharan Africa: acupuncture, herbal products, massage therapy, meditation, mind-body practices, prayer, reflexology, spiritual healing, supplement use (with and without dietary modifications), various traditional medicines (African, Chinese and Indian origin), vitamins/minerals and yoga.

Since 2000, the WHO has been encouraging African nations and other low- and middle-income countries to embrace the use of traditional and complementary medicines by incorporating it into their national health systems in an attempt to improve the access to affordable healthcare (WHO 2002). However, developing a national policy and legal framework dealing with T&CM is still a great challenge for several sub-Saharan African nations because of the lack of evidence regarding its effectiveness, risks and interactions.

Patients using T&CM with poor understanding or based on misleading information may present a risk to their well-being (Mwaka, Abbo & Kinengyere 2020). In the same light, healthcare providers with insufficient knowledge about the mechanism of action of T&CM, their safety profiles, side effects and possible interactions with conventional medicines would be unable to improve the health of the general population who are T&CM users (Mwaka et al. 2020).

The prevalence of cancer is increasing worldwide, especially in SSA. Additionally, most of the cancer patients from SSA are being diagnosed in advanced stages where survival rates are poor, often prompting these patients to turn to the use of T&CM (Mwaka et al. 2020). There are, however, limited data available on aspects of complementary medicine use among cancer patients in SSA, including the types of T&CM used, effectiveness and risks of such treatments and the extent to which cancer patients use or rely on T&CM (Mwaka et al. 2020).

A systematic review, conducted by James et al. (2018), found that most patients using T&CM, whether alone or in combination with mainstream medicine, do not disclose the use of T&CM to their physicians, either because their physicians do not ask about T&CM use or the patient fears retribution. When it comes to anticancer drugs, most have a very narrow therapeutic window, and the use of herbal supplements, which a cancer patient may perceive as harmless, could interact with their chemotherapy regimen reducing its efficacy or enhancing its toxic effects (Vasques et al. 2024). It is therefore of great importance that patients disclose any T&CM use to their oncologist.

This scoping review aims to collect information about the prevalence of use, reasons for use, most common types of T&CM used, level of satisfaction with T&CM and disclosure of T&CM use to physicians among cancer patients in sub-Saharan Africa. The collection and reporting of information regarding T&CM used by patients with cancer will hopefully guide other researchers on future investigations that can be done to fill knowledge gaps; encourage more research to be conducted to determine the mechanisms of action of T&CM products, their indications, effectiveness, side effects from direct use, and interactions between T&CM and conventional treatments.

## Research methods and design

### Study design

This scoping review is aimed at collating existing knowledge on T&CM use and identifying gaps in knowledge regarding T&CM use among adult cancer patients in SSA. This scoping review was done using the Arksey and O’Malley methodological framework (2005); the following five key steps were followed: (1) specifying the research question, (2) identifying relevant literature or studies, (3) study selection, (4) charting of data and (5) collating, summarising and reporting of results. Studies written in English, published in the last 10 years, and addressing the use of T&CM among cancer patients in SSA were reviewed.

This scoping review will focus on studies conducted in SSA. While T&CM practices may vary across different cultures, many regions tend to share similar medical practices and beliefs (James et al. 2018). Owing to the limited number of studies conducted on T&CM use among cancer patients, this study region will allow for conclusions and averages to be drawn for the area, even though most studies may emerge from a few countries. A timeframe of 10 years was chosen to ensure the review reflects the most current data available on popular T&CM being utilised by cancer patients.

#### Step 1: Specifying the research question

In order to collate existing knowledge and identify gaps in knowledge regarding T&CM, the aim of this scoping review is to collect evidence on the prevalence of use, reasons for use, common types of T&CM used, level of satisfaction with T&CM and disclosure of T&CM use with physicians among cancer patients in sub-Saharan Africa.

The following five sub-questions were formulated to shape the goals and outcomes of this review:

- To what extent are studies conducted assessing the prevalence of T&CM use among adult cancer patients in sub-Saharan African countries?
- To what extent are studies conducted assessing the reasons why adult cancer patients use T&CM in sub-Saharan African countries?
- To what extent are studies conducted assessing the different types of T&CM used by adult cancer patients in sub-Saharan African countries?
- To what extent are studies conducted assessing the level of satisfaction with T&CM use among adult cancer patients in sub-Saharan African countries?
- To what extent are studies conducted assessing the portion of adult cancer patients who disclose T&CM use with their physician in sub-Saharan African countries?

#### Step 2: Identifying relevant studies

*Inclusion criteria*: Studies that fulfilled the following criteria were included in the review:

- Published article or study has been peer-reviewed.
- Published between 01 January 2013 and 31 December 2022.
- Published in the English language.
- The research article focuses on the use of T&CM by adult cancer patients (above 18 years) with any type of cancer.
- Cancer patients participating in the study self-reported T&CM use while undergoing any form of conventional cancer therapy.
- Data were collected from a country (or countries) in sub-Saharan Africa.
- Study outcome included the prevalence of T&CM use and other aspects of T&CM use that are related to cancer.

*Exclusion criteria*: Studies were excluded if they were:

- Published before 01 January 2013 or after 31 December 2022.
- Articles or studies with data collected from patients outside of sub-Saharan Africa.

*Search strategies*: Available articles were gathered, reviewed and organised from the different databases: PubMed, ScienceDirect, Google Scholar and EBSCOhost from 2013 to 2022. The following keywords were used in the electronic database searches: ‘complementary medicine’, ‘alternative medicine’, ‘traditional medicine’, ‘complementary therapy’, ‘alternative therapy’, ‘traditional therapy’, ‘complementary treatment’, ‘alternative treatment’, ‘traditional treatment’, ‘African traditional medicine’, ‘herbal medicine’, ‘herbal supplements’, ‘medicinal plants’, ‘indigenous therapy’, ‘use’, ‘types’, ‘prevalence’, ‘attitude’, ‘knowledge’, ‘pattern’, ‘practice’, ‘perception’, ‘utilization’, ‘health seeking behaviour’, ‘cancer patients’, ‘sub-Saharan Africa’, ‘SSA’, ‘Angola’, ‘Benin’, ‘Botswana’, ‘Burkina Faso’, ‘Burundi’, ‘Cape Verde’, ‘Cameroon’, ‘Central African Republic’, ‘Chad’, ‘Comoros’, ‘Congo’, ‘Democratic Republic of Congo’, ‘Cote d’Ivoire’, ‘Djibouti’, ‘Equatorial Guinea’, ‘Eritrea’, ‘Ethiopia’, ‘Gabon’, ‘Gambia’, ‘Ghana’, ‘Guinea’, ‘Guinea-Bissau’, ‘Kenya’, ‘Liberia’, ‘Madagascar’, ‘Malawi’, ‘Mali’, ‘Mauritania’, ‘Mauritius’, ‘Mozambique’, ‘Namibia’, ‘Niger’, ‘Nigeria’, ‘Rwanda’, ‘Sao Tome and Principe’, ‘Senegal’, ‘Seychelles’, ‘Sierra Leone’, ‘Somalia’, ‘South Africa’, ‘South Sudan’, ‘Sudan’, ‘Swaziland’, ‘Tanzania’, ‘Togo’, ‘Uganda’, ‘Zambia’, ‘Zimbabwe’. The Boolean operators ‘and’ and ‘or’ were used when necessary to narrow or expand the search, respectively. An example of how search terms were used to conduct the search in each data base can be seen in Table 1-A1.

#### Step 3: Study selection

**FIGURE 1:**
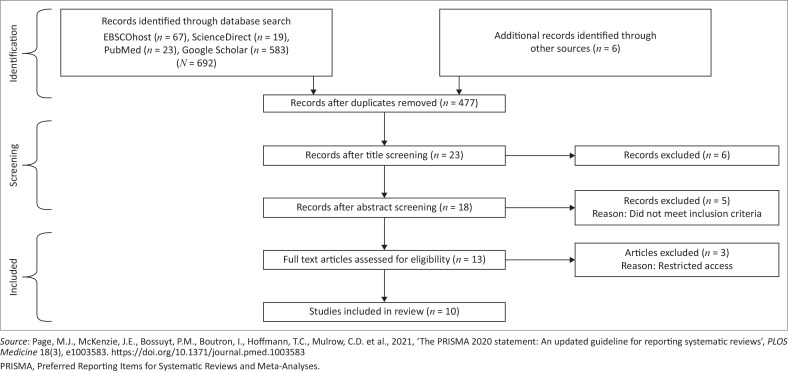
PRISMA flow diagram outlining the study selection process.

The relevant studies identified from the various databases were gathered and duplicates were removed. Thereafter, screening of titles and abstracts was performed to assess eligibility and relevance to the goals of the scoping review. Articles that passed abstract screening underwent full-text analysis. Those studies that met the necessary criteria and were considered eligible were included in the scoping review. The PRISMA (Preferred Reporting Items for Systematic Reviews and Meta-Analyses) flowchart (Figure 1) outlines the study selection process.

#### Step 4: Charting of data

The charting method was used for data analysis (Levac, Colquhoun & O’Brien 2010). Data were extracted from the articles and stored in a Microsoft Word table drawn up with the headings necessary to answer the research question and record all relevant information sought by the scoping review. The template included the following headings: ‘Title of article’, ‘First author and Year of publication’, ‘Type of study design’, ‘Country’, ‘Prevalence of use among cancer patients’, ‘Reasons for use’, ‘Reported satisfaction with T&CM’, ‘Disclosure of T&CM’ and ‘Types of T&CM used’. This format made it easy to compare the outcomes of each study and identify which aspects were not covered (see Table 2-A1).

#### Step 5: Collating, summarising and reporting of results

The results were collated according to sub-topics in order to best meet the aim of the scoping review and answer each aspect of the research question. The eligible articles were summarised using an outcome-based descriptive analysis. Statistical analyses of the data collected from the articles were not conducted because of the heterogeneity of each study’s intent and design. Only the median and average were calculated for all aspects, and their respective ranges are reported in Table 3-A1.

### Ethical considerations

An application for full ethical approval was made to the Biomedical Research Ethics Committee of University of KwaZulu-Natal, and ethics consent was received on 11 October 2023. The ethics approval number is BREC/00005784/2023. All procedures performed in studies involving human participants were in accordance with the ethical standards of the institutional and/or national research committee and with the 1964 Helsinki Declaration and its later amendments or comparable ethical standards. Written informed consent was obtained from all individual participants involved in the study.

## Results

Sub-Saharan Africa consists of the 49 countries in the southern part of the African continent (Tapon 2023). Articles included in the scoping review only contained information from eight countries in sub-Saharan Africa (Ghana, Ethiopia, Nigeria, Malawi, Tanzania, Morocco, Uganda and Kenya). Conclusions were drawn based on articles analysed, thus only covering 16.3% of sub-Saharan Africa.

The use of T&CMs ranged from 14.1% to 84%. Average T&CM use is 66.7% based on the 10 articles included in the scoping review. The country with the highest T&CM use among cancer patients was Malawi (84%) and the country with the lowest T&CM use by cancer patients was Kenya (14.1%).

### Reasons for traditional and complementary medicine use among cancer patients

Nine out of the 10 articles included described reasons why cancer patients opted to use T&CM. From five of the articles, it was found an average of 45.3% of cancer patients stated that the reason they are using T&CM is based on the belief that it can cure cancer. The study conducted in Kenya reported the highest percentage of cancer patients using T&CM to cure cancer (64%), while the study conducted in Morocco revealed the lowest percentage (4%) of use to cure cancer, while the other four articles did not mention patients using T&CM to cure cancer. Traditional and complementary medicine being used in order to improve psychological well-being was evaluated in four articles. The average percentage of patients using T&CM for psychological comfort is 38.1%. The use of T&CM based on the belief that it boosts the body’s immunity was covered in four articles. An average of 31.6% of T&CM users stated the reason for T&CM use was to boost immunity. From the 10 articles included, 5 articles addressed cancer patients using T&CM for symptomatic relief – on average more than a quarter of cancer patients (26.7%) used some form of T&CM to treat cancer symptoms.

An average of 66.5% of cancer patients reported using T&CM as they believed it to increase their quality of life. According to two articles, an average of 52.1% of patients used T&CM as they were willing to try anything or were simply being hopeful (Ong’udi et al. 2019; Yarney et al. 2013).

Two articles made mention that a portion of patients used T&CM as they were disappointed or dissatisfied with conventional cancer treatment. Cancer patients using T&CM for this reason were stated to be 9.4% in Ghana (Yarney et al. 2013) and 14.9% in Ethiopia (Erku 2016).

Other reasons why cancer patients used T&CM were because of lack of cancer services, fear of surgical operations, spiritual and cultural importance, conventional treatment being expensive and to reduce side effects of chemotherapy.

### Types of traditional and complementary medicine used

When analysing the common types of T&CM used by cancer patients, all 10 articles mentioned the use of herbs and herbal medication. The use of herbal products ranged from a minimum of 22% in Uganda to a maximum of 91% among Kenyan cancer patients who were T&CM users. The second most common type of T&CM reported by patients, and covered in six articles, was prayer and spiritual/faith/traditional-based practices. Cancer patients who reported using prayer ranged from 8.5% to 80.3% and those using spiritual/faith/traditional healing ranged from 8.2% to 64%. Seven articles addressed dietary changes and supplement use; an average of 20.3% of T&CM users made diet modifications such as an increase in vegetables, or fruits, or fish and/or meat, or milk or a decrease in sugar and began taking supplements. Each of the following categories was mentioned in four different articles with the average use in brackets: vitamins/minerals (37%), massage (32.7%) and Chinese medicine (20.1%). Each of the following categories were covered in two different articles with the average use in brackets: spiritual sacrifice (11.4%), acupuncture (2.8%), mind-body practices (2.5%), yoga (9.3%) and meditation (15.3%). Reflexology (3.8%), Indian medicine (5%) and scarification (10%) were also only covered once.

### Reported satisfaction with traditional and complementary medicine

Patient satisfaction with T&CM was described in different ways in the articles. Nine of the 10 articles addressed patient satisfaction with T&CM in some way or another. Some articles described the believed benefits of T&CM according to the cancer patients using them, while other articles described which types of T&CM they found beneficial, or simply stated the percentage of users who were satisfied with complementary therapy. From the nine articles, the following benefits were mentioned, and the portion of T&CM users who saw these benefits is stated in brackets: fought cancer (40.6%), reduced the severity of cancer (23.2%), improved sleep (17.4%), improved emotional and physical well-being (14.5%), reduced nausea and vomiting (11.9%), improved appetite (10.1%) and reduced pain (6.9%). From the study done in Malawi, 88% of cancer patients who made dietary changes saw improvements and were satisfied. From those who used faith-based healing, 50% satisfaction was reported. An average of 72% reported contentment with vitamin/mineral supplements and 16% with herbal medicine. Four articles stated the percentage of cancer patient T&CM users who were satisfied with complementary therapy use in general; satisfaction ranged from 22% to 54.5%.

### Disclosure of traditional and complementary medicine to physician

Seven out of the 10 articles reported the percentage of T&CM users that informed their physicians regarding their use of complementary treatment. An average of 21.2% of cancer patients disclosed T&CM use to their medical doctor. The portion of patients who told their physicians about T&CM use ranged from 5% in Morocco to 55% in Kenya. Some of the reasons why cancer patients did not tell their physicians about T&CM use include the following: assuming doctors have a negative response to T&CM, patients think it is not important for doctors to know about T&CM use, physicians do not ask about T&CM, patients refuse to tell their doctors, and patients are afraid that oncologists might discourage T&CM use.

## Discussion

It is evident that the use of T&CM is common among those diagnosed with cancer in SSA, and the varying prevalence of T&CM use in different countries can be associated with each region’s accessibility to conventional medicine, sociocultural backgrounds and the importance placed on T&CM by the general population. Additionally, increased use of T&CM over the years is evidenced in two studies conducted in Enugu, Nigeria, among gynaecologic cancer patients. The study by Nwankwo et al. published in 2019 found that over a 2-year period (June 2014 to June 2016), 64.3% of gynaecologic cancer patients were T&CM users (Nwankwo et al. 2019), and the study conducted by Ajah et al. published in 2021 found 71.5% of participants made use of T&CM (Ajah et al. 2021).

The study conducted in Ethiopia by Erku (2016) found that the most common source of T&CM information was family members and friends, and the least common source used to seek information about T&CM was medical practitioners. The study done in Nigeria and Ghana reported similar findings in which most of the participants got information about T&CM from friends and family or mass media (Yarney et al. 2019). The study done in Tanzania found that more than half of respondents used herbal medicine for their current cancer disease; however, only a quarter consulted a traditional healer or herbalist (Henke, Bruchhausen & Massawe 2022). In the study done in Nigeria by Nwankwo et al. (2019), it was also revealed that most of the T&CM being used were self-administered and no professional herbalists were consulted; the products being used were mainly recommended by family and friends. A large population of T&CM users sourced products informed by friends, family or the media and do not seek help from herbalists or physicians. This presents a great challenge as herbal agents are prone to abuse among T&CM users as they are not counselled about how to use them; furthermore, it could be a potentially toxic compound when combined with anticancer treatment (Nwankwo et al. 2019).

Several cancer patients stated the reason they were using T&CM was to cure cancer. Owing to the important role traditional healers have held in the region and the large population who still have strong belief in traditional healers, it is important for healers and physicians to work together in order to deliver the best possible care to patients. Many cancer patients believe that traditional medicine has the ability to cure cancer; it is therefore crucial that physicians remind patients that traditional healing does not replace conventional anticancer treatment and all forms of T&CM need to be used with caution.

Anticancer agents are generally toxic with several side effects. Some cancer patients tend to combine herbal products with their conventional cancer treatment in an attempt to enhance well-being and manage the side effects associated with chemotherapy. However, the potential risk of interactions from herbal use may outweigh any benefit (Alnaim 2007). For example, a case report by Bilgi et al. (2010) reported that a patient taking imatinib for 7 years began displaying symptoms of hepatotoxicity upon starting to consume ginseng. Once ginseng was discontinued, the hepatotoxicity resolved. According to the study conducted by Cox et al. (2006), garlic was found to decrease the clearance of docetaxel. Despite the decrease being non-statistically significant, there is a possibility of increased adverse effects resulting from docetaxel accumulation. The pharmacokinetic study by Goey et al. (2014) concluded that St John’s wort caused a remarkable decrease in the plasma concentration of docetaxel.

In a Nigerian study, more than a third of the participants stated they used T&CM with the belief it would complement the conventional medicine and improve the outcome of treatment. A quarter of respondents also stated that they used T&CM because it was readily available and one fifth found it more affordable. This can be expected as a large portion of the African population rely on T&CM to meet their primary medical needs (Aliyu et al. 2017).

The study conducted among cancer patients in Uganda found that those patients who are dissatisfied with conventional treatment are more likely to use T&CM (Kiwanuka 2018). Respondents also stated that they turned to T&CM use in order to fulfil unmet needs by conventional therapy (Kiwanuka 2018). The growing use of traditional and herbal-based medicinal therapies may be because of the perception that they are natural and therefore do not cause any detrimental or unfavourable effects despite the lack of scientific evidence (Erku 2016).

A study conducted in Ghana found that approximately one-third of cancer participants used T&CM because they were willing to try anything. In the developing world (including Ghana), a large portion of cancer patients are diagnosed at a late stage, where many conventional treatments often fail to cure the individual (Yarney et al. 2019). The late diagnosis leads to reduced chances of cure; thus, many patients lose hope in the effectiveness of conventional treatment and turn to using or relying on traditional, complementary and alternative medicines (Yarney et al. 2019).

More than half of the cancer patients who participated in the study in Malawi used herbal medicines, even though the percentage of patients who consulted with herbalists was lower. The following herbs were commonly being reported: Terasi or Teras Juice, mvunguti (*Kigelia africana*), ginger (*Zingiber officinale*), moringa (*Moringa oleifera*), garlic (*Allium sativum*), aloe vera, avocado leaves (*Persea americana*) and soursop (*Annona muricata*). Herbal products are particularly important when looking into the impact T&CM has on cancer treatment (potential herb–drug interactions, patients delaying conventional therapies, toxic herbal products). Another problem is that a significant proportion of herbal product users state that they are often unaware of which herbs they are consuming. It was also found that many herbalists do not disclose the herbs that they supply in order to maintain proprietary rights to their herbal formulations and prevent patients from self-administering herbal products (Hill et al. 2022).

From the articles included, it was noticed that many participants reported adverse effects such as nausea, vomiting, frequent urination, diarrhoea, headache, upset stomach, skin rashes and itching.

The study conducted in Malawi by Hill et al. published in 2022 revealed relatively low satisfaction with herbal products and traditional healers. Several participants conveyed dissatisfaction because of the inability to formally diagnose cancer, control their symptoms or cure the cancer. It is common for patients to first go to traditional healers when they experience symptoms and only turn towards mainstream healthcare after traditional methods were unable to produce desired results. This may contribute to the dissatisfaction with traditional methods upon receiving a pathological cancer diagnosis (Hill et al. 2022).

Less than a quarter of cancer patients disclosed T&CM use with their medical doctor. The main reason why participants did not inform their physician about T&CM use was because their doctor did not ask. Some of the additional reasons why patients do not disclose T&CM use to their physicians are because cancer patients believe their physicians would not understand T&CM usage, disapprove of their use of T&CM or force them to discontinue T&CM treatment. The fact that a large portion of cancer patients would not disclose that they are using T&CM until they are asked makes it important for every oncologist to ask their patients whether they are using T&CM, what products they use, how frequently they use them, and the manner in which they use them. The large portion of cancer patients who believe their physicians will have a negative attitude towards their T&CAM use also highlights the need for T&CM use sensitivity training so the topic can be addressed in a safe and comfortable way with patients.

It is important for T&CM users to remember that some of the information they receive about T&CM are often based on opinions and personal experiences, not based on objective research studies. The use of natural products is commonly associated with being safer than conventional treatment; however, this is not essentially correct. Guidance from nurses and healthcare professionals is key to help patients distinguish between possible deceit and evidence-based natural products. Patients who solely believe traditional healers can cure their cancer often delay or stop standard treatment, resulting in the reduced chances of remission or cure. It is of vital importance for healthcare providers to familiarise themselves with the most commonly used T&CM treatments among cancer patients so they will be equipped to answer questions about T&CM and advise patients in the best manner possible, warning them about over-stated benefits and potential harms of a particular therapy (Mwaka et al. 2020).

### Limitations

This scoping review may have omitted relevant studies that could have been in another language or to which full access was not available. The presence of methodological flaws in the research design of included studies would reduce the accuracy of reported results. All studies included in the review are cross-sectional studies; however, they vary in the manner T&CM was addressed and categorised, thus potentially affecting the reporting of results.

## Conclusion

From the review, the authors can conclude that many cancer patients in SSA use at least one form of T&CM during their cancer diagnosis. Reasons for using T&CM vary among patients but the most common reasons include to improve physical and psychological well-being, to cure cancer or treat symptoms, to boost immunity, to improve quality of life and because they believe in its benefits. It was found that many patients used various T&CM products concurrently with conventional anticancer treatment; additionally, very few patients disclosed the use of complementary medicine to their physician. This non-disclosure of T&CM use exposes cancer patients to potentially dangerous side effects from interactions between drugs and T&CM products. Healthcare providers need to start having more open discussions on T&CM use with their patients in order to prevent abuse of T&CM products and provide them with factual information on T&CM safety and efficacy.

There is a need for more scientific research to be conducted in order to understand pharmacodynamics and pharmacokinetics of T&CM products to successfully determine their therapeutic indications, safety and risk profiles. This will also assist policymakers who are working on integrating T&CM and conventional biomedical treatments. The inclusion of T&CM in the medical school curricula can improve the knowledge and attitudes of healthcare workers regarding T&CM use by their patients.

## Appendix 1

**TABLE 1-A1:** Example of Search Strategy completed.

Database, search date	Search terms	Number of articles retrieved after title screening
PubMed, 3 April 2023	‘Complementary Medicine’ OR ‘Alternative Medicine’ OR ‘Traditional Medicine’ OR ‘Complementary Therapy’ OR ‘Alternative Therapy’ OR ‘Traditional Therapy’ OR ‘Complementary Treatment’ OR ‘Alternative Treatment’ OR ‘Traditional Treatment’ OR ‘African Traditional Medicine’ OR ‘Herbal Medicine’ OR ‘Herbal Supplements’ OR ‘Medicinal Plants’ OR ‘Indigenous Therapy’ AND ‘use’ OR ‘types’ OR ‘prevalence’ OR ‘attitude’ OR ‘knowledge’ OR ‘pattern’ OR ‘practice’ OR ‘perception’ OR ‘utilization’ OR ‘health seeking behaviour’ AND ‘Cancer Patients’ AND ‘Sub Saharan Africa’ OR ‘Sub-Saharan Africa’ OR ‘SSA’ OR ‘Angola’ OR ‘Benin’ OR ‘Botswana’ OR ‘Burkina Faso’ OR ‘Burundi’ OR ‘Cape Verde’ OR ‘Cameroon’ OR ‘Central African Republic’ OR ‘Chad’ OR ‘Comoros’ OR ‘Congo’ OR ‘Democratic Republic of Congo’ OR ‘Cote d’Ivoire’ OR ‘Djibouti’ OR ‘Equatorial Guinea’ OR ‘Eritrea’ OR ‘Ethiopia’ OR ‘Gabon’ OR ‘Gambia’ OR ‘Ghana’ OR ‘Guinea’ OR ‘Guinea-Bissau’ OR ‘Kenya’ OR ‘Liberia’ OR ‘Madagascar’ OR ‘Malawi’ OR ‘Mali’ OR ‘Mauritania’ OR ‘Mauritius’ OR ‘Mozambique’ OR ‘Namibia’ OR ‘Niger’ OR ‘Nigeria’ OR ‘Rwanda’ OR ‘Sao Tome and Principe’ OR ‘Senegal’ OR ‘Seychelles’ OR ‘Sierra Leone’ OR ‘Somalia’ OR ‘South Africa’ OR ‘South Sudan’ OR ‘Sudan’ OR ‘Swaziland’ OR ‘Tanzania’ OR ‘Togo’ OR ‘Uganda’ OR ‘Zambia’ OR ‘Zimbabwe’ AND published between 1 January 2013 and 31 December 2022	14 (Total records identified = 23)

**TABLE 2-A1:** Summary of data extracted from selected articles.

Title of article	First author and year of publication	Type of study design	Country	Prevalence of use among cancer patients	Reasons for use	Reported satisfaction with T&CM	Disclosure of T&CM	Types of T&CM used
Characteristics of users and implications for the use of complementary and alternative medicine in Ghanaian cancer patients undergoing radiotherapy and chemotherapy: A cross-sectional study	Yarney et al. (2013)	Cross-sectional, mixed-method research design	Ghana	73.5% of the 98 patients surveyed used CAM	Try anything = 31.2% Faith/beliefs = 21.9% Sickness is spiritual = 15.6% Toxicity of conventional treatment = 9.4% Conventional doctors are mechanical to patient = 12.5% Disappointed with conventional treatment = 9.4%	Patient-stated benefits: fight cancer (40.6%), relieve severity of the cancer (23.2%), relaxation or sleep (17.4%), improve emotional and physical well-being (14.5%) Adverse effects: gastric upset, nausea and vomiting, diarrhoea, itching, headaches	16.7% of T&CM users informed their physicians.	Massage = 66.3% Herbal = 59.2% Mega vitamins = 55.1% Chinese medicines = 53.1% Prayer = 42.9%
Complementary and alternative medicine use and its association with quality of life among cancer patients receiving chemotherapy in Ethiopia: A cross-sectional study	Erku (2016)	Cross-sectional, mixed-method research design	Ethiopia	79.0% of 195 participants used CAM	Belief in advantages = 73.4% Dissatisfaction with conventional therapy = 14.9% Family tradition = 13.0% Emotional support = 11.0% Boost immunity = 8.4%	49.3% reported satisfaction with use. 9.7% were dissatisfied with use. 81.8% of users reported no adverse effects from T&CM.	20.8% of T&CM users informed their physicians. 56.5% assumed doctors have negative response for T&CM use. 22.1% thought it was not important for doctors to know about T&CM use.	Herbs = 72.1% Special foods = 38.9% Spiritual healing = 36.4% Dietary supplements = 22.1%
Prevalence and correlates of complementary and alternative medicine use among cancer patients in Usmanu Danfodiyo University Teaching Hospital, Sokoto, Nigeria	Aliyu et al. (2017)	Cross-sectional, mixed-method research design	Nigeria	66.3% of 240 participants were T&CM users	T&CM improves treatment outcome with conventional medications = 37.1% Readily available = 25.1% T&CM use has spiritual and cultural importance = 18.9% More affordable =18.9%	Benefits: Reduce nausea and vomiting = 11.9% Improves appetite = 10.1% Reduces pain = 6.9% 64.2% reported adverse effects. Nausea and vomiting = 52.5%. Diarrhoea = 44.2%	Physicians were unaware in 87.4% of cases. Reason: Doctor did not ask = 45.3% Refusal to tell doctor = 43.2%	Prayer = 30.8% Herbal therapy = 28.3% Aloe vera = 10%, Nutritional therapy = 3.1% Forever living products = 13.8% Scarification = 10% Indian medicine = 5%
Prevalence of traditional, complementary, and alternative medicine (TCAM) among adult cancer patients in Malawi	Hill et al. (2022)	Cross-sectional, mixed-method research design	Malawi	84% out of 263 patients use T&CM. 60% combined a T&CM with conventional cancer. 40% used conventional medicine alone	Majority of participants used T&CM to directly treat cancer versus for symptom management	Percentage of highest satisfaction (very satisfied) attributed to: Diet change = 88%, Vitamins/minerals = 72% Faith-based healing = 50% Herbal medicine = 16%	Not reported	Faith-based healing = 64% Herbal medicine = 56% Diet change = 46% Vitamins/minerals = 23%
Complementary and alternative medicine use among gynaecological cancer patients in Enugu, South-East Nigeria	Ajah et al. (2021)	Cross-sectional, mixed-method research design	Nigeria	71.5% of the 396 respondents used CM	Presumed it will cure the cancer = 62.8% For symptom relief = 37.1%	30.4% of CM users stated benefit.	84.5% of doctors were unaware of patients’ use of CAM.	Herbs = 65% Combined prayers with herbs = 13.8 % Spiritual sacrifice = 9.5% Prayer = 8.5% Medicinal = 3.2%
Use of herbal medicine is associated with late-stage presentation in Tanzanian patients with cancer: A survey to assess the utilisation of and reasons for the use of herbal medicine	Henke et al. (2022)	Cross-sectional, mixed method research design	Tanzania	70% of 302 patients surveyed use Herbal Medicine for their current cancer disease	It may cure cancer = 45% Symptom relief = 16% Lack of cancer services in their area = 15% Traditional reasons = 11% Recommended by others = 4% Reduce side effects of chemotherapy = 4% Expensive = 2% Fear of operation = 1%	Poor level = 73.7% Good satisfaction = 24.4% Excellent satisfaction = 1.4%	77.5% of users did not tell their oncologists.	Types of herbal medicine used not reported
Prevalence of complementary and alternative medicine use in patients with haematological malignancies in Marrakech, Morocco	Tazi et al. (2013)	Cross-sectional, mixed-method research design	Morocco	68% out of the 184 patients interviewed used CAM	Improve physical well-being or increase quality of life = 51% Boost immune system = 32% Relieve cancer symptoms = 13% Treat or cure cancer = 4%	Saw no benefit = 78% Could describe specific benefits = 22% Transient side effects = 4% (related to ingesting herbs) Side effects included nausea, headache, diarrhoea	95% of T&CM users did not inform their doctors. Most common reason: Haematologists might have discouraged T&CM use (87%).	65% used T&CM techniques and 72% used T&CM substances. Plants = 39% (*Nigella* and *Aristolochia longa* = 30%) Pure honey = 32% ZamZam water = 3% Religious practices = 58% Traditional healers = 9% Special diets = 4%
Complementary and alternative medicine use and challenges among gynaecological cancer patients in Nigeria: Experiences in a tertiary health institution	Nwankwo et al. (2019)	Cross-sectional, mixed-method research design	Nigeria	64.3% of 95 participants	Cure cancer = 50.8% Stop vaginal bleeding = 31.2% Enhancing body’s immunity = 13.1% Improve psychological well-being = 4.9%	21.3% of T&CM users experienced at least one side effect	Not reported	Herbs = 73.8% Spiritual sacrifice = 13.2% Diet modification = 5.3% Chinese medicine = 4.9% Prayers = 44.3%
Complementary and alternative medicine use: influence of patients’ satisfaction with medical treatment among breast cancer patients at Uganda Cancer Institute	Kiwanuka (2018)	Cross-Sectional, mixed method research design	Uganda	77% of 235 female breast cancer patients use CAM	Not reported	Not reported (Respondents not satisfied with medical treatment were more likely to use T&CM therapies [odds ratio, 2.146])	Not reported	Herbal = 22.0%, Prayer = 20.0%, Vitamins = 13.8%, Native healers = 8.2%, Chinese medicines = 5.5%, Massage = 4.4%, Yoga = 2.7%, Ayurvedic medicine = 1.6%, Acupuncture = 3.3%, Reflexology = 3.8%, Attending support group = 3.8%, meditation = 2.2%, Magnetic and Bio-field manipulation = 1.1%
Study of the use of complementary and alternative medicine by cancer patients at Kenyatta National Hospital, Nairobi, Kenya	Ong’udia et al. (2019)	Cross-sectional, mixed-method research design	Kenya	14.1% out of 78 patients used CAM	Restore hope = 73.0% Psychological comfort = 82.0% Increase life quality = 82.0% Boost immunity = 73.0% Cure their disease = 64% Symptoms relief = 36%	54.5% of users were satisfied with T&CM use 55.0% were disappointed with use 27.0% of users reported side effects (vomiting and frequent urination)	55% of T&CM users disclosed use to their physician.	Herbs = 91.0% Faith healing = 54.5% Divination = 36.3% Massage = 27.3%

T&CM, traditional and complementary medicine; CAM, complementary and alternative medicine.

**TABLE 3-A1:** Data collated from eligible articles.

Aspects evaluated across included articles	Number of articles that explored mentioned aspect	Range	Median	Mean
Prevalence of T&CM use among cancer patients	10	84−14.1 = 69.9	8.0	66.7
**Reasons for use**				
Willing to try anything/hope	2	73–31.2 = 41.8	52.1	52.1
Faith/belief/tradition	3	21.9–11 = 10.9	13.0	15.3
Believe in T&CM advantages	1	-	73.4	73.4
Sickness is spiritual, T&CM is of spiritual importance	2	18.9–15.6 = 3.3	17.3	17.3
Dissatisfied with conventional treatment	2	14.9–9.4 = 5.5	12.2	12.2
Side effects of conventional treatment	2	9.4–4 = 5.4	6.7	6.7
Improves emotional/psychological well-being	3	82–4.9 = 77.1	32.8	38.1
Boost immunity	4	73–8.4 = 64.6	22.6	31.6
More affordable	2	18.9–2 = 16.9	10.5	10.5
Readily available	2	25.1–15 = 10.1	20.1	20.1
T&CM improves conventional treatment outcomes	1	-	37.1	37.1
Cure or treat cancer	5	64–4 = 60	50.8	45.3
Symptom relief	5	37.1–13 = 24.1	31.2	26.7
Increase quality of life	2	82–51 = 31	66.5	66.5
Recommended by others	1	-	4.0	4.0
**Patient Reported Satisfaction Regarding T&CM**				
Satisfaction stated with T&CM use in general	4	54.5–22 = 32.5	39.9	39.1
Dissatisfied/poor level	3	73.7–9.7 = 64	55.0	46.1
Good satisfaction	1	-	24.4	24.4
Excellent satisfaction	1	-	1.4	1.4
Reported side effects	5	64.2–4 = 60.2	21.3	26.9
Fought cancer/relieved severity	2	40.6–23.2 = 17.4	31.9	31.9
Improve emotional and physical well-being	1	-	14.5	14.5
Improve sleep	1	-	17.4	17.4
Reduce nausea and vomiting	1	-	11.9	11.9
Improves appetite	1	-	10.1	10.1
Reduces pain	1	-	6.9	6.9
Vitamins/mineral	1	-	72.0	72.0
Traditional/faith-based healing	1	-	50.0	50.0
Diet change	1	-	88.0	88.0
Herbal medicine	1	-	16.0	16.0
Portion of cancer patients who disclosed T&CM use to physicians	7	55–5 = 50	16.7	21.2
**Reasons for nondisclosure**				
Patient assumed doctor has negative attitude towards T&CM	1	-	56.5	56.5
Did not think it was important to inform doctor	1	-	22.1	22.1
Doctor did not ask	1	-	45.3	45.3
Refusal to tell doctor	1	-	43.2	43.2
Doctor might discourage T&CM use	1	-	87.0	87.0
**Types of T&CM used by cancer patients**				
Herbal products	10	91–22 = 69	62.1	57.7
Vitamins/minerals	4	56.2–13.8 = 42.4	39.1	37.0
Chinese medicines	4	53.1–4.9 = 48.2	12.1	20.1
Indian medicine	1	-	5.0	5.0
Ayurvedic medicine	1	-	1.6	1.6
Prayer	6	58–8.5 = 49.5	36.9	34.1
Dietary modifications/supplement use	7	46–3.1 = 42.9	22.1	20.3
Faith/spiritual/traditional healing	6	64–8.2 = 55.8	36.4	34.7
Spiritual sacrifice	2	13.2–9.5 = 3.7	11.4	11.4
Aloe vera	2	10–3.3 = 6.7	6.7	6.7
Pure honey	1	-	32.0	32.0
ZamZam water	1	-	3.0	3.0
Forever living products	1	-	13.8	13.8
Scarification	1	-	10.0	10.0
Mind-body practices	2	2.7–2.2 = 0.5	2.5	2.5
Massage	4	66.3–4.4 = 61.9	30.1	32.7
Acupuncture	2	3.3–2.2 = 1.1	2.8	2.8
Yoga	2	15.8–2.7 = 13.1	9.3	9.3
Meditation	2	28.4−2.2 = 26.2	15.3	15.3
Reflexology	1	-	3.8	3.8

T&CM, traditional and complementary medicine.
